# iTRAQ-Based Quantitative Proteomic Analysis Identified HSC71 as a Novel Serum Biomarker for Renal Cell Carcinoma

**DOI:** 10.1155/2015/802153

**Published:** 2015-09-03

**Authors:** Yushi Zhang, Yi Cai, Hongyan Yu, Hanzhong Li

**Affiliations:** Department of Urology, Peking Union Medical College Hospital, Chinese Academy of Medical Sciences and Peking Union Medical College, Beijing 100730, China

## Abstract

Renal cell carcinoma (RCC) is one of the most lethal urologic cancers and about 80% of RCC are of the clear-cell type (ccRCC). However, there are no serum biomarkers for the accurate diagnosis of RCC. In this study, we performed a quantitative proteomic analysis on serum samples from ccRCC patients and control group by using isobaric tag for relative and absolute quantitation (iTRAQ) labeling and LC-MS/MS analysis to access differentially expressed proteins. Overall, 16 proteins were significantly upregulated (ratio > 1.5) and 14 proteins were significantly downregulated (ratio < 0.67) in early-stage ccRCC compared to control group. HSC71 was selected and subsequently validated by Western blot in six independent sets of patients. ELISA subsequently confirmed HSC71 as a potential serum biomarker for distinguishing RCC from benign urologic disease with an operating characteristic curve (ROC) area under the curve (AUC) of 0.86 (95% confidence interval (CI), 0.76~0.96), achieving sensitivity of 87% (95% CI 69%~96%) at a specificity of 80% (95% CI 61~92%) with a threshold of 15 ng/mL. iTRAQ-based quantitative proteomic analysis led to identification of serum HSC71 as a novel serum biomarker of RCC, particularly useful in early diagnosis of ccRCC.

## 1. Introduction

Renal cell carcinoma (RCC) is the most frequent form of kidney cancer, with an increasing incidence over the past decades [1]. The majority of RCC are of the clear-cell type (ccRCC), which accounts for approximately 80% of kidney cancer [2]. Early diagnosis of RCC is one of the most important factors contributing to the successful treatment and favorable prognosis. The diagnosis of RCC is mainly based on imaging findings, which however has limited accuracy and cannot be used reliably to confirm the nature of the lesion [3]. Due to the increase of disease rates, together with the fact that there are no serum biomarkers available, inexpensive and noninvasive test of prediction for RCC would be urgently required for the early detection of RCC. Serum and plasma, containing proteins both secreted and shed from tumor cells, are ideal fluids for the detection of cancer biomarkers since they are characterized by ease of sampling and storing. However, their variable composition and vast dynamic range of proteins present in serum pose tremendous technical challenges in identifying clinically relevant biomarkers [4]. A recently novel proteomics named isobaric tags for relative and absolute quantification (iTRAQ) combined with mass spectrometry technology (LC-MS/MS) now represents a powerful tool for identification of cancer biomarkers [5]. The iTRAQ technology has been successfully applied to biomarker screening of multiple tumors and diseases in both tissue and serum samples [6].

In this study, we performed quantitative proteomic analysis using the iTRAQ and LC-MS/MS to identify proteins dysregulated in serum of early-stage ccRCC patients compared to healthy people. We revealed differential expression of a number of proteins in serum of ccRCC patients compared with control group. In addition, we confirmed the most dysregulated expression of heat shock cognate 71 kDa protein (HSC71) on six independent sets of serum by Western blot. ELISA subsequently confirmed HSC71 as a potential serum biomarker for diagnosis of RCC.

## 2. Materials and Methods

### 2.1. Patients and Serum Collection

This study was approved by the Human Ethics Committee of Peking Union Medical College Hospital. Serum samples from healthy volunteers and patients were all collected from the Department of Urology at Peking Union Medical College Hospital between October 2013 and October 2014. The control group consisted of 10 healthy controls and 20 patients diagnosed with other urologic diseases such as angiomyolipoma of kidney (10 patients), benign prostatic hyperplasia (4 patients), urinary tract infection (4 patients), and urolithiasis (2 patients). The detailed demographic profiles of the participants are provided in Table 1. All samples were collected before breakfast, then centrifuged at 3000 g for 15 min at 4°C, and subsequently stored at −80°C prior to further processing. All serum samples were collected before any treatment or surgery.

### 2.2. Affinity Depletion of Serum Samples

To reduce the individual differences, sera collected were pooled into two groups, each containing sera from 10 RCC patients or 10 healthy people. Pooled serum samples were depleted of the 14 high-abundance proteins using a high capacity 4.6 × 100 mm multiple affinity removal column (Agilent Technologies, CA). Approximately 35 *μ*L of serum was processed per sample and analyzed with the Agilent 1260 HPLC system according to the manufacturer's protocol. Protein concentrations were determined using a Bradford protein assay kit (Pierce). All measurements were performed in duplicate.

### 2.3. iTRAQ Labeling and Strong Cation Exchange Liquid Chromatography

Prior to iTRAQ labeling, the samples were concentrated and desalted using 10 kDa molecular weight cutoff spin concentrators (Millipore). Then, 100 *μ*g peptides from each sample were labeled with the iTRAQ reagents according to the manufacturer's instructions and as previously described [7]. Peptides were labeled individually with iTRAQ tag (Applied Biosystems, USA) as follows: 114.1 for ccRCC and 115.1 for control group, respectively. To reduce sample complexity, labeled samples were pooled and fractionated by strong cation exchange (SCX) using a Polysulfoethyl A column (PolyLC Inc., USA) as previously described [7].

### 2.4. Reverse-Phase LC-MS/MS Analysis

Dried SCX fractions were reconstituted in 100 mL of buffer A (5% acetonitrile; 95% H_2_O; 0.1% formic acid) and loaded into a reverse-phase C18 Peptide Captrap (Agilent Technologies, USA). After desalting, peptides were eluted by running a 5% to 80% buffer B gradient (95% acetonitrile; 5% H_2_O; 0.1% formic acid) at a flow rate of 0.4 *μ*L/min for 65 min. The LC elution was subjected to positive-ion nanoflow electrospray analysis using a Qstar XL MS/MS system (Applied Biosystems Inc., USA). A survey scan was acquired from* m/z* 400–1800 for 0.5 s with up to four precursor ions selected from* m/z* 100–2000 for MS/MS. Each fraction from SCX chromatography was analyzed in duplicate.

### 2.5. Western Blot Analysis

The 14 most abundant proteins were depleted from sera from 6 RCC patients and 6 healthy people using a high capacity 4.6 × 100 mm multiple affinity removal column. Total protein concentration was determined with Bio-Rad Protein Assay Dye Reagent Concentrate (Bio-Rad, USA). Protein samples (10 *μ*g) were separated on a 10% SDS-PAGE gel, transferred to PVDF membranes, and probed with rabbit polyclonal antibodies to HSC71 (R&D, USA) overnight at 4°C. As there are no reliable internal control proteins for Western blot analysis of the serum samples, a loading control sample was generated by pooling all of the samples from all groups in equal volumes (20 *μ*L) for each gel. The optical intensity of each protein staining was determined using Quantity One. The loading control sample in each gel was used as the standard for quantification.

### 2.6. ELISA Analysis

Human heat shock cognate 71 kDa protein ELISA kit from Huamei Biological Inc. (Wuhan, China) was purchased and used according to the manufacturer's instruction. The serum levels of HSC71 were assayed in the 30 RCC patients, 10 healthy controls, and 20 other urologic diseases patients. All serum samples and the standards were run in duplicate.

### 2.7. Statistical Data and Graphics

We performed the entire statistical analysis with SPSS 19.0. Student's *t*-test was applied for comparisons of quantitative data. We performed operating characteristic curve (ROC) analysis to quantify serum HSC71 positivity and statistical uncertainty. Data are expressed as the mean ± standard deviation (M ± SD). For all analysis, a *P* value < 0.05 was considered to indicate statistical significance.

## 3. Results

### 3.1. Quantitative Proteomic Analysis of Serum Proteins by iTRAQ

To identify dysregulated serum proteins between ccRCC patients and control group, we compared 10 ccRCC patients' sera and 10 healthy people's sera by iTRAQ quantitative proteomic analysis. Among 375 identified proteins, 16 proteins were increased more than 1.5-fold and 14 proteins were decreased less than 0.67-fold in the serum of early-stage ccRCC patients compared to that of healthy controls (Table 2). Furthermore, serum HSC71 was highly elevated compared to control group (3.07-fold upregulated expression) and was evaluated further for its potential as a serum biomarker for ccRCC.

### 3.2. Validation of Serum HSC71 Upregulation by Western Blot

**Figure 1:**
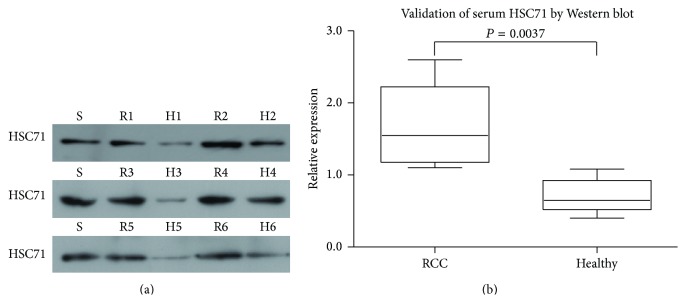
Validation of the serum HSC71 by Western blotting. (a) Serum HSC71 level was further examined using Western blotting in 6 additional serum samples from ccRCC patients (R1–R6) and healthy people (H1–H6). (b) Quantification of the densitometric analysis of the Western blotting bands was performed. The average concentration of HSC71 was 2.39-fold (*P* = 0.0037) higher in the RCC groups than in the Healthy group.

To confirm the dysregulated expression of HSC71 in ccRCC patients and healthy controls sera, we performed Western blot analysis in six sets of discovery cohort individually (Figure 1(a)). Quantitative analysis showed that HSC71 was significantly upregulated in sera of ccRCC (*P* = 0.0037) (Figure 1(b)), in agreement with our iTRAQ quantitative proteomic findings.

### 3.3. Validation of Serum HSC71 as a Novel Biomarker for ccRCC by ELISA

**Figure 2:**
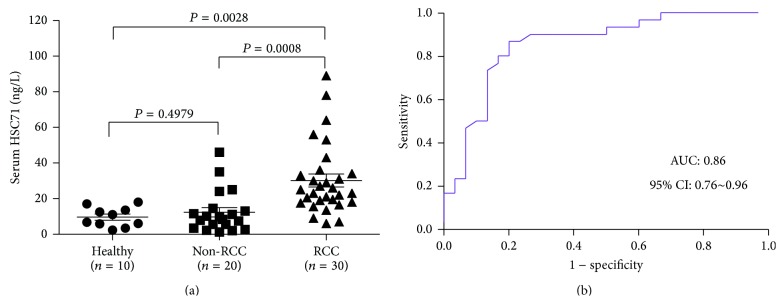
Validation of the diagnostic value of serum HSC71 concentration in RCC by ELISA. (a) The serum HSC71 concentration in 30 RCC patients, 10 healthy people, and 20 other urologic diseases (Non-RCC) was determined using ELISA. The mean concentrations of serum HSC71 in Healthy, Non-RCC, and RCC groups were 9.64, 12.34, and 30.17 ng/L, respectively. Both Healthy and Non-RCC groups showed significantly lower levels of HSC71 than the RCC group (*P* = 0.0028, *P* = 0.0008). (b) The receiver-operating characteristic curve (ROC curve) for the serum levels of HSC71 in patients with RCC compared with control group. The area under each curve is 0.86 (95% CI, 0.76~0.96).

ELISA, performed in 30 ccRCC patients, 10 healthy people, and 20 patients with other urologic diseases (Table 1), showed significantly higher serum HSC71 levels in ccRCC patients versus the control group (10 healthy people and 20 other urologic diseases patients) (Figure 2(a)). These observations were consistent with the results obtained by iTRAQ analysis and Western blot, and the HSC71 concentrations obtained by ELISA were used to assess the clinical performances of the serum HSC71. The diagnostic values of serum HSC71 were evaluated by ROC curve analysis. The area under the receiver-operating characteristic curve (AUC) for differentiating between ccRCC and control group was 0.86 (95% confidence interval (CI), 0.76~0.96), achieving sensitivity of 87% (95% CI 69%~96%) at a specificity of 80% (95% CI 61~92%) with a threshold of 15 ng/mL (Figure 2(b)).

## 4. Discussion

In this study, we initially used two pooled samples for proteomic studies to identify the biomarker candidates using iTRAQ and LC−MS/MS. HSC71 was strongly increased in the sera samples from the RCC patients compared to control group. Then, we examined serum HSC71 levels of six sets of screening samples by Western blotting individually and confirmed that the data were comparable with quantitative proteomic analysis. Finally, we examined the clinical significance of HSC71 by ELISA in 30 RCC patients, 20 other urologic disease patients, and 10 healthy people to evaluate the clinical utilities of the candidate with ROC curve analysis. We demonstrated for the first time that serum HSC71 concentration is a biomarker for the diagnosis of RCC.

HSC71, also known as heat shock 70 kDa protein 8 (HSPA8), is a member of the heat shock protein 70 family, located on chromosome 11q23.3. It is constitutively expressed under nonstressful conditions and also participated in protein folding as well as differentiation procedures [8, 9]. Previous studies showed that upregulation of HSC71 in leukemic cells contributes to cell cycle disruption, and HSC71 binding to cyclin D1 in nuclei leads to the stabilization of the cyclin D1/CDK4 complex, promoting cell proliferation [10]. High levels of HSP70 family expression have been demonstrated in human cancers, which appear at least of prognostic value in osteosarcoma [11, 12]. In the previous studies, heat shock proteins (HSPs) family expression is induced in a variety of kidney diseases suggesting that specific HSPs may have distinct functions in renal malignancies [13]. However, to our knowledge, no studies have evaluated the value of serum HSC71 in the potential diagnosis role for RCC.

Here we report raised HSC71 concentrations in sera from RCC patients compared with healthy people and other urologic diseases. HSPs have gained interest as a promising anticancer drug target, due to its importance in maintaining the stability, integrity, conformation, and function of key oncogenic proteins, including HSP40, HSP70, and HSP90 family members [14, 15]. In addition, regarding the differentially expressed proteins, some of these were previously reported to be involved in RCC such as serum albumin [16], haptoglobin [17], and alpha-1-acid glycoprotein 1 [18], which provides confidence to our dataset and provides an independent confirmation of these candidates. In addition, previous studies have reported that prostate cancer, colon cancer, and squamous cell lung cancer may secrete common plasma proteins such as zinc-alpha-2-glycoprotein [19, 20] and complement C9 [21]. However, those candidate biomarkers need further research to confirm the clinical utility of as biomarker for diagnosis of RCC.

Clinical applications of serum HSC71 may require additional investigation. First, our results were obtained only for pT1-pT3 RCC patients; therefore, more pT4 and metastasis RCC patients should be included in the further validation studies. In addition, we could not show statistically significant correlation between serum HSC71 levels and different stages of RCC patients because of small patient numbers. The potential association of serum HSC71 with progression of RCC or tumor load requires further investigation using a larger sample. Second, the optimal cutoff value should be determined in a larger number of serum samples. Any single marker may not be sufficient for the final decision about therapeutic strategy, and all clinical and pathological information should be considered when using biomarker data. We do not consider that HSC71 is the only and best biomarker protein for RCC and the additional biomarker candidates should be further investigated using the other proteomic modalities.

## 5. Conclusion

In conclusion, using iTRAQ-based comparative proteomic analysis and validating by Western blotting and ELISA analysis, we identified serum HSC71 concentration as a novel biomarker for the diagnosis of RCC. Further investigation of the origin of serum HSC71 and the mechanisms underlying the correlation between serum HSC71 and carcinogenesis will allow greater insights into RCC biology.

## Figures and Tables

**Table 1:** Description and comparison of clinical and laboratory characteristics of the study subjects.

Characteristic	Discovery samples by iTRAQ		Validation samples by ELISA	
	RCC (*n* = 10)	Control (*n* = 10)	RCC (*n* = 30)	Control (*n* = 30)
Age (years)	54.80 ± 13.32	52.80 ± 9.13	53.10 ± 13.33	55.63 ± 13.98
Gender (male/female)	7/3	7/3	18/12	16/14
BMI	22.86 ± 2.57	22.55 ± 2.55	21.78 ± 2.83	22.16 ± 3.02
Hypertension	4 (40%)	2 (20%)	6 (20%)	4 (13.3%)
Diabetes mellitus	2 (20%)	1 (10%)	5 (16.7%)	3 (10%)
Smoking	3 (30%)	3 (30%)	7 (23.3%)	6 (20%)
Pathological stage				
pT1	10 (100%)		16 (53.3%)	
pT2			11 (36.7%)	
pT3			2 (6.7%)	
pT4			1 (3.3%)	
M0	10 (100%)		28 (93.3%)	
M1			2 (6.7%)	
Fuhrman grades				
G1-2	10 (100%)		21 (70%)	
G3-4			9 (30%)	
Histological subtype				
Clear-cell	10 (100%)		23 (76.7%)	
Papillary			3 (10%)	
Chromophobe			4 (13.3%)	

Data are expressed as *n* (%), or mean ± SD. RCC: renal cell carcinoma.

**Table 2:** List of differentially expressed proteins in ccRCC compared to control group.

Accession	Description	Score	Coverage	Ratio
Proteins upregulated in ccRCC compared with control group				
P11142	Heat shock cognate 71 kDa protein	31.11	4.80%	3.07
P02763	Alpha-1-acid glycoprotein 1	251.48	40.80%	2.087
Q16777	Histone H2A type 2-C	164.19	49.61%	1.984
P68871	Hemoglobin subunit beta	320.13	60.54%	1.942
Q00532	Cyclin-dependent kinase-like 1	20.04	1.96%	1.828
P02748	Complement C9	984.63	41.86%	1.815
P02787	Serotransferrin	1689.45	61.46%	1.775
P07360	Complement component C8 gamma chain	466.53	61.39%	1.753
P02768	Serum albumin	2997	82.76%	1.705
P69905	Hemoglobin subunit alpha	140.62	26.76%	1.697
P25311	Zinc-alpha-2-glycoprotein	275.61	35.91%	1.673
P01031	Complement C5	2686.11	49.16%	1.646
P68363	Tubulin alpha-1B chain	127.08	6.21%	1.605
P00738	Haptoglobin 1 alpha	922.06	59.61%	1.58
Q12931	Heat shock protein 75 kDa	43.68	1.99%	1.546
Q8NG11	Tetraspanin-14	34.11	2.96%	1.518

Proteins downregulated in ccRCC compared with control group				
Q96KN2	Beta-Ala-His dipeptidase	142.84	12.62%	0.651
Q5T686	Arginine vasopressin-induced protein 1	45.27	4.08%	0.643
P01614	Ig kappa chain V-II region Cum	83.17	33.04%	0.627
P55056	Apolipoprotein C-IV	189.85	40.94%	0.622
Q16610	Extracellular matrix protein 1	643.87	34.44%	0.614
Q96KK5	Histone H2A type 1-H	177.42	50.00%	0.606
P14618	Pyruvate kinase PKM	47.37	3.95%	0.582
P33908	Mannosyl-oligosaccharide	60.74	4.75%	0.495
P01019	Angiotensinogen	620.93	28.45%	0.426
O60814	Histone H2B type 1-K	67.21	15.87%	0.419
Q15166	Serum paraoxonase/lactonase 3	138.75	11.86%	0.415
P68431	Histone H3.1	24.05	5.15%	0.377
P01815	Ig heavy chain V-II region COR	12.87	5.83%	0.37
P62805	Histone H4	171.24	45.63%	0.353
